# Look to Norway: Serving new families and infants in a multiethnic population

**DOI:** 10.1002/imhj.21804

**Published:** 2019-07-18

**Authors:** Maria J. Leirbakk, Jeanette H. Magnus, Johan Torper, Paula Zeanah

**Affiliations:** ^1^ Department of Health Sciences University of Oslo Norway and City of Oslo Agency for Health; ^2^ Section for Leadership University of Oslo, Oslo Norway and Tulane School of Public Health and Tropical Medicine New Orleans Louisiana USA; ^3^ Department for Health and Social Services City of Oslo Norway; ^4^ Picard Center, College of Nursing University of Louisiana Lafayette Louisiana USA

**Keywords:** home visits, infant mental health, MCH service, public health nurses, salutogenesis, servicio MCH, visitas a casa, enfermeras de salud pública, salutogénesis, salud mental infantil, Servie MCH, visites à domicile, infirmières de santé publique, salutogénèse, santé mentale du nourrisson, MCH‐Service, Hausbesuche, Pflegekräfte, Salutogenese, psychische Gesundheit von Säuglingen, 母子保健サービス, 家庭訪問, 保健師, 健康生成論, 乳幼児精神保健, MCH服務, 家訪, 公共衛生護士, 致敬理論, 嬰兒心理健康, خدمة صحة الأم والطفل، الزيارات المنزلية، ممرضات الصحة العامة، التكوين الصحي، الصحة النفسية للرضع

## Abstract

Despite recognition that immigrant women face significant health challenges, addressing the healthcare needs of immigrants is a source of debate in the United States. Lack of adequate healthcare for immigrants is recognized as a social justice issue, and other countries have incorporated immigrants into their healthcare services. Oslo, the fastest growing capital in Europe, is rapidly shifting to a heterogeneous society prompting organizational action and change. The New Families Program serves first‐time mothers and their infants in an Oslo district serving 53% minorities from 142 countries. Anchored in salutogenic theory, the program aims to support the parent–child relationship, children's development and social adaptation, and to prevent stress‐related outcomes. Formative research has informed the successful program development and implementation within the existing maternal and child healthcare service. Implications for addressing maternal and child health needs of an immigrant population are presented.

## RESUMEN

A pesar de reconocerse que las mujeres inmigrantes enfrentan significativos retos en cuanto a salud, las discusiones sobre las necesidades de cuidado de salud de inmigrantes es fuente de debate en Estados Unidos. La falta de un adecuado sistema de cuidado de salud para inmigrantes es identificado como un asunto de justicia social y otros países han incorporado a los inmigrantes dentro de sus servicios de cuidado de salud. Oslo, la capital europea con mayor crecimiento, está pasando rápidamente a ser una sociedad heterogénea lo cual conlleva acción y cambios organizacionales. El programa las Nuevas Familias ayuda a madres primerizas y sus infantes en un distrito de Oslo que sirve a un 53% de grupos minoritarios de 142 países. Basado en la teoría salutogénica, el programa se propone apoyar la relación madre‐niño, el desarrollo y la adaptación social de los niños, así como prevenir resultados relacionados con el estrés. La investigación formativa es la base del éxito del desarrollo e implementación del programa dentro del existente servicio de cuidado de salud materno e infantil. Se presentan las implicaciones para discutir las necesidades de salud materno‐infantiles de una población inmigrante.

## RÉSUMÉ

Bien qu'il soit reconnu que les femmes immigrées font face à des défis de santé importants, le fait de considérer les besoins de santé des immigrés est une source de débat aux Etats‐Unis. Le manque de soins de santé pour les immigrés est reconnu comme étant une question de justice sociale et d'autres pays ont incorporé les immigrés dans leurs services médicaux. Oslo, en Norvège, la capitale européenne qui grandit le plus vite, est rapidement en train d’évoluer vers une société hétérogène exigeant une action organisationnelle et des changements. Le programme des Nouvelles Familles sert des mères étant mères pour la première fois et leurs nourrissons dans une partie de la ville d'Oslo qui contient 53% de minorités venues de 142 pays. Ancré dans une théorie de salutogénèse, ce programme a pour but de soutenir la relation parent‐enfant, le développement des enfants et leur adaptation sociale, et de prévenir des résultats liés au stress. Des recherches formatives ont influencé le succès du développement du programme ainsi que sa mise en place au sein d'un service de soins maternels et de l'enfant existant déjà. Les implications concernant l'approche des besoins de santé pour les mères et les enfants de cette population immigrée sont présentées.

## ZUSAMMENFASSUNG

Ungeachtet der Erkenntnis, dass Migrantinnen mit erheblichen gesundheitlichen Herausforderungen konfrontiert sind, ist die Adressierung der Bedürfnisse von Einwanderern ein Diskussionsthema in den USA. Der Mangel an angemessener Gesundheitsversorgung für Einwanderer gilt als Problem der sozialen Gerechtigkeit, und andere Länder haben Einwanderer in ihre Gesundheitsdienste aufgenommen. Oslo, die am schnellsten wachsende Hauptstadt Europas, wandelt sich zunehmend in eine heterogene Gesellschaft, die organisatorisches Handeln und Veränderungen erfordert. Das „New Families Program“ richtet sich an Erstmütter und deren Kleinkinder in einem Osloer Stadtteil, der 53% der Minderheiten aus 142 Ländern einschließt. Verankert in der Theorie der Salutogenese, zielt das Programm darauf ab, die Eltern‐Kind‐Beziehung, die kindliche Entwicklung und soziale Anpassung zu unterstützen und stressbedingte Folgen zu verhindern. Die Grundlagenforschung hat die erfolgreiche Entwicklung und Umsetzung des Programms innerhalb des bestehenden Gesundheitssystems für Mütter und Kinder geprägt. Es werden Implikationen für das Adressieren der gesundheitlichen Bedürfnisse von Müttern und Kindern einer immigrierten Bevölkerung vorgestellt.

## 抄録

ノルウェーを見つめて: 多民族集団における新しい家族と乳幼児にサービスを提供する

移民女性は著しい健康に関する難問に直面しているにもかかわらず、アメリカ合衆国において、移民の健康管理の必要性に取り組むことは議論の素である。移民の十分な健康管理の欠如は、社会的公正の問題とみなされ、他国では、移民はその地の健康管理サービスに組み入れられる。ヨーロッパにおいて最も人口が増えている首都オスロは、急速に組織の行動と変化を促す多元社会へと変化しているところである。ニュー・ファミリープログラムは、初めての母親とその乳幼児に行なわれるもので、オスロ地域では142の国から来た53％の少数派に実施されている。健康生成理論に基づいて、プログラムは親子関係、子どもの発達、社会的適応を支援し、ストレス関連の結果を予防するのがねらいである。基礎調査は、現存する母子保健管理サービス内での成功したプログラムの進歩と実行を報じた。移民層の母子保健の必要性に取り組むことの意味を述べる。

## 摘要

看看挪威:為多民族人口提供新的家庭和嬰兒服務

儘管認識到移民婦女面臨重大的健康挑戰, 但解決移民的醫療保健需求在美國仍有爭論。移民缺乏足夠的醫療保健被認為是一個社會公義問題, 其他國家已將移民納入其醫療保健服務。奧斯陸是歐洲發展最快的首都, 正在迅速變成多元化的社會, 促使組織行動和改變。新家庭計劃為奧斯陸地區的首次母親及其嬰兒提供服務, 為來自142個國家的53％少數民族提供服務。該計劃以致敬理論為基礎, 旨在支持親子關係丶兒​​童發展和社會適應, 並預防與壓力相關的結果。這個形成性研究, 為現有的母嬰保健服務中的成功項目開發和實施提供了信息。並討論解決移民人口的母嬰健康需求。

## ملخص

نظرة إلى النرويج: خدمة الأسر الجديدة والرضع في مجتمع سكني متعدد الأعراق

على الرغم من الاعتراف بأن المهاجرات يواجهن تحديات صحية كبيرة، فإن تلبية احتياجات الرعاية الصحية للمهاجرين هي مصدر نقاش في الولايات المتحدة، ويُعترف بعدم توفر الرعاية الصحية الكافية للمهاجرين كقضية عدالة اجتماعية، وهناك بلدان أخرى أدمجت المهاجرين في خدمات الرعاية الصحية الخاصة بهم. على سبيل المثال أوسلو، وهي أسرع عاصمة نموا في أوروبا، تتحول بسرعة إلى مجتمع غير متجانس يدفع إلى اتخاذ إجراءات تنظيمية وتغيير. يخدم برنامج الأسر الجديدة لأول مرة الأمهات وأطفالهن الرضع في منطقة أوسلو التي تخدم 53% من الأقليات من 142 دولة. ويهدف البرنامج، الذي يرتكز على نظرية الصحة، إلى دعم العلاقة بين الوالدين والطفل، ونمو الأطفال والتكيف الاجتماعي، ومنع النتائج المتعلقة بالإجهاد. وقد استرشدت البحوث التكوينية بنجاح تطوير وتنفيذ البرامج في إطار خدمات الرعاية الصحية للأمهات والأطفال القائمة. وتعرض الدراسة التطبيقات التي تتناول تلبية احتياجات صحة الأم والطفل في مجتمع المهاجرين.

## 1 INTRODUCTION

Rapid change stresses all segments of the government, including the healthcare system, creating an escalating demand for service. Yet, despite recognition of the unique healthcare needs of immigrant women and children (American Academy of Pediatrics [AAP], 2005; American College of Obstetrics and Gynecologists [ACOG], 2015) as well as recognition that barriers to sexual and reproductive healthcare for women, and the health of their children, are human rights and social justice issues (Lauen, Henderson, White, & Kohchi, 2017; World Health Organization, 2015), addressing the healthcare needs of immigrants is a source of much debate in the United States. Other countries have adopted an accepting approach and taken political actions to incorporate immigrants into their healthcare services, and Norway is an example of this approach. Oslo is the fastest growing capital in Europe (22.3%/10 years) (World Population Review, 2016). The city's rapid shift to a heterogeneous society with substantial variations in educational attainment, tradition, health literacy, and financial means creates challenges across the health and social services. In this article, Norway's example of developing and providing services to first‐time mothers and their infants in an Oslo district serving 53% minorities from 142 countries is described. The long‐term aims of the New Families Program are to improve parent and child relationships, child development, children's social adaptation, school readiness, and possibly reducing costly downstream public health measures. Use of formative research, community participation, professional and community education, and critical reflection have contributed to the successful implementation and expansion of the existing maternal and child public health services program. Based on the initial pilot, the New Families Program has been further developed and implemented as a citywide program. The development and implementation of this program provide an example of how maternal and infant health and mental health needs can be met when social and reproductive justice considerations are embedded within the health and social system of the country. However, before describing Norway's approach, we will briefly reflect on the policy in the United States regarding immigrant healthcare.

## 2 IMMIGRANT HEALTHCARE IN THE UNITED STATES

Developed with the priorities of reunification of families, admitting workers with skills that are valuable to the U.S. economy, protection for those who face persecution in their homeland, and insuring diversity, especially from underrepresented countries (Congressional Budget Office, 2006, p. vii) (American Immigration Council, 2016), U.S. immigration policies and procedures are complicated, confusing, controversial, and undergoing change. The United States has seen a 70% increase in the percentage of immigrants of the U.S. population since 1995, from 9% (24.5 million) to 13% (42.3 million) in 2014. Including first (foreign‐born) and second (children of foreign‐born) generations, immigrants account for 25% of the U.S. population. These numbers include an estimated 11.4 million undocumented immigrants in the United States (Zong & Batalova, 2017).

Healthcare access for immigrants in the United States depends on a variety of factors, including immigration status, date of immigration, type of program or service needed, and state or jurisdiction in which the immigrant lives. In most cases, immigrant's access to healthcare is less than that for U.S. citizens (Ku & Jewers, 2012). Interestingly, a “healthy migrant effect” finds that immigrants often have better health when they enter the United States, but their health declines because immigrants have less access to resources such as healthcare and adopt unhealthy U.S. lifestyles (e.g., diet, smoking, substance use; Fennelly, 2005).

Pregnant women and children present unique challenges. Immigrant women are more likely to be poor, less likely to access prenatal care, and have greater risk of poor pregnancy and infant outcomes (ACOG, 2015). Children are at risk for numerous health and developmental problems as well (AAP, 2005). Many undocumented immigrant women and children have faced violence and abuse, trafficking, and/or high levels of stress related to their immigration experience (ACOG, 2015; Futures Without Violence, n.d.). However, undocumented immigrants are excluded from services provided through the Patient Protection and Affordable Care Act (2010) including Medicare and Medicaid (with exceptions), although they may access health services through Federally Qualified Health Centers (FQHCs), migrant health centers, free clinics, and emergency care via the Emergency Medical Treatment and Active Labor Act (1985) (ACOG, 2015; Perreira et al., 2012). Since access to services depends on the status of the individual, some members of “mixed‐status” families may be eligible for service and others not, and families often face numerous practical and social barriers to accessing services.

From a reproductive justice standpoint, U.S. immigration policies are particularly challenging for women and children. Reproductive justice provides an organizing framework and approach that seeks to ensure that “all people have the economic, social, and political power and means to make decisions about their bodies, sexuality, health, and family, with dignity and self‐determination” (http://latinainstitute.org/en/what-we-do/immigrant-women, n.d.). The impact of social determinants of health such as human rights and social equity highlights the complexity of health issues for immigrant women and their families (Davies, Basten, & Frattini, 2006). Finally, the importance of good pregnancy health and positive support for the emerging parent–infant relationship underscores the long‐ranging implications of immigrant healthcare for infant mental health.

To better address the healthcare needs of a vulnerable immigrant maternal–child population, it is useful to examine how other countries with different policies do so. In 1942, during World War II, President Franklin D. Roosevelt stated

If there is anyone who still wonders why this war is being fought, let him look to Norway. If there is anyone who has any delusions that this war could have been averted, let him look to Norway; and if there is anyone who doubts the democratic will to win, again I say, let him look to Norway. (Warbey, 1945, p. 2)

Thus, we look to Norway's example of care for vulnerable immigrant mothers and children.

## 3 IMMIGRANTS IN NORWAY

Historically, heterogeneity of the population in the United States has been at a stark contrast to Norway. For years, Norway has been deemed the very best country in which to live (United Nations Development Programme, 2016), and it tops the World Economic Forum's Inclusive Development Index of the world's most advanced and developed economy based on indicators from poverty to public debt and environmental factors (Corrigan, 2017). Paradoxically, for the past century, Norway has had a high degree of social disparity. Oslo is the capital in Europe with the largest degree of health disparity. The diversity is primarily based on socioeconomic status, education, and health literacy. Some districts with high socioeconomic status and well‐educated citizens have a life expectancy of 8 to 12 years longer than do districts with low socioeconomic status and low education (Statistics Norway, 2016a).

Like the United States, the influx of immigrants into Norway has resulted in a growth of immigrants and their descendants from 1% of the population in the 1970s to over 16% in 2016 (Statistics Norway, 2016b). Norway is not part of the European Union, but labor mobility is one of the main reasons for the extensive influx of migrants because Norway is part of the European Economic Area (EEA). Norway also experiences an increased influx of refugees, asylum seekers, and undocumented immigrants, resulting in new demands on the Norwegian healthcare system. Children of undocumented immigrant parents born in Norway are not automatically a Norwegian citizen. The Gross National Product allocation to the healthcare system per capita has increased substantially in the last decades, and was 12% in 2016 (Statistics Norway, 2016a). The increase in immigration and the clustering of immigrants in certain counties, especially in Oslo, accentuate a dire need for holistic measures to tackle the social disparities and the additional challenges brought by the recent peak of immigration.

### 3.1 Norwegian health policies

The Norwegian system provides salient contrasts to the U.S. system of care. While both value a decentralized approach and free choice of provider, Norway emphasizes the principle of universal access, and the good health of workers and the population is regarded as a national security responsibility. Healthcare is financed by taxation, together with income‐related employee and employer contributions as well as out‐of‐pocket copayments. All residents, including migrants, labor immigrants from EEA, legal immigrants, refugees, or asylum seekers under assessment, are covered by the National Insurance Scheme (Folketrygden, NIS), managed by the Norwegian Health Economics Administration (Helseøkonomiforvaltningen). Similar to the United States, if an application for asylum in Norway is refused, the general healthcare rights are lost (discussed later). However, everyone living in Norway has the right to emergency care. In addition, *all pregnant women* in Norway, no matter legal status, have rights to free abortion, prenatal care, and care related to delivery, and the immediate follow up at the Maternal and Child Health Care Service (MCHS). Furthermore, *all children* up to age 18 years, no matter their legal status, have the right to free preventive services provided by public health nurses (PHNs) and regular clinical examinations by general practitioners (GPs) (discussed later). Contraceptives are free of charge and available at pharmacies for all girls between 16 to 18 years of age, and at reduced cost up to age 20 years. The PHNs or midwives can prescribe contraceptives to older women. Condoms are free and can be ordered from the Norwegian Health Directorate and delivered by mail to the home address (https://www.gratiskondomer.no/bestill/) no matter the legal status of the recipient. While healthcare policy is controlled centrally, responsibility for the provision of healthcare is decentralized. In Norway, all community‐based public health and social welfare services, including primary care, the MCHS, school health services, nursing homes, and home care for the elderly and disabled, are integrated and under the jurisdiction of the local county or city health administration. These services are funded by allocation of tax income to each municipality and district. All Norwegian citizens are invited to choose their GP from a list, and 99% of them do so. These GPs, outpatient doctors, are the core of the Norwegian Primary Health Care Services and act as gatekeepers for specialized care, including mental health. Use of private medical insurance is limited in Norway, but has grown some the last 20 years.

Immigrants and asylum seekers who are granted residence permits are expected to participate in free Norwegian language and culture classes. The intention is not to change their culture but rather to teach them about the culture that their new country practices and to familiarize them with the laws. Family members granted permanent residences through family reunion are not required to participate but are offered the classes. Unfortunately, this policy has led to low participation of female family members, with the consequence of a lack of language knowledge and comprehension, limited ability to understand and participate in the society, and poor follow‐up of their children in school. When children with a home language other than Norwegian are school‐ready, the law obligates the school to teach in the home language of the child if the family so prefers. In some schools, as many as 25 different languages are offered. However, the family often prefers the child to learn only Norwegian in addition to the obligatory English that starts in first grade in all Norwegian schools.

### 3.2 Primary healthcare services (PHCS)

Local authorities at the municipal level organize and finance PHCS according to local demand. The services are available for everyone, but the degree of copayment depends on legal status. There is, however, a free Health Centre for Undocumented Immigrants in Oslo, which is a drop‐in service, based on the work of volunteer health professionals. As already mentioned, children under the age of 18 have the same rights to health services as do Norwegian children, and pregnant women have access to prenatal maternity care, free delivery, postnatal care, and abortion through the MCHS.

The MCHS is considered a core service of the Norwegian healthcare system, and includes preventive services for infant, preschool, and school services for all children no matter their legal status up to age 18 years. This is in addition to an extensive program for reproductive rights for women, including during pregnancy, childbirth, and postpartum. All families receive one home visit by the PNH and one home visit by the midwife when the child is a newborn, and each infant receives monthly clinic‐based checkups of growth and development during the first year of life. Between ages 1 and 4 years, 5 of the 14 recommended visits at the MCHS are scheduled according to the immunization program, with multiple vaccines administered by the PHN; 5 of these visits include a routine development assessment and clinical examination of the child by a GP. Close to 100% of all children attend and follow the prescribed pattern of MCHS visits. After age 4, the school health nurse follows the child from the age of 5 to 20. The ideological underpinning of the service is to prevent diseases and accomplish continuing good health status of all children, and the main underlying principle of the MCHS is to improve parents’ autonomy and independence. Norwegian White Papers have described the mother–child service as the basis of the wider public health establishment (Ministry of Social Affairs, 1992–1993). Thus, in addition to the provision of primary healthcare, the PHN is charged with the responsibility of reporting “inadequate” and harmful parenting to the Child Protection Service (Andrews, 2003). Notably, Norwegian PHNs complete a 1‐year certificate in public health nursing after finishing their baccalaureate nursing program (Glavin, Schaffer, Halvorsrud, & Kvarme, 2014).

### 3.3 Migrant health in Norway

Equal rights are enshrined in Norwegian legislation enabling immigrants (referred to as *documented immigrants*) and asylum seekers to access healthcare and medical treatment. However, refused asylum seekers and undocumented immigrants waiting to be expelled have only free‐of‐charge access to emergency services and the MCHS services. The number of undocumented immigrants in Norway is unknown, but is estimated to be 20,400 (0.4% of the population). In Norway, 80% of undocumented immigrants are refused asylum seekers, and the remaining 20% are anticipated to be guest workers with expired licenses who did not return to their country (Øien, Sønsterudbråten, 2011).

In Norway, reproductive rights are supported, prenatal care is followed up, and all care related to the pregnancy and delivery is free for all. Nevertheless, these women have additional healthcare needs. For example, a prospective study has demonstrated that depression in pregnancy (defined as an Edinburgh Perinatal Depression Scale score of ≥10; Cox, Holden, & Sagovsky, 1987) was twice as prevalent in ethnic minority women, especially in Middle Eastern and South Asian women, as compared to Western Europeans, even after adjusting for known risk factors (Shakeel et al., 2015). In addition, gestational diabetes and weight gain during pregnancy are more prevalent in the immigrant population (Sommer et al., 2015).

In Norway, immigrants have less multimorbidity (Diaz et al., 2015), commonly defined as the presence of two or more chronic medical conditions in an individual. Interestingly, multimorbidity is highest among refugees at arrival, but increases rapidly among labor immigrants, especially in women (Diaz et al., 2015). Adult documented immigrants in Norway have a lower utilization of the PHCS. This might reflect better health among them, but it also could be due to barriers to access PHCS (Diaz & Kumar 2014). A recent review of studies has concluded that immigrants in Norway have a higher burden and a greater risk for mental health problems than do Norwegians in the general population (Abebe, Lien, & Hjelde, 2014). This higher risk, specifically among adult immigrants from low‐income, non‐Western countries, is associated with social and economic deprivation, negative life events pre‐ and postmigration, and lack of social support. There are limited data regarding unaccompanied minors, but the Norwegian Institute of Public Health published results from a longitudinal study on unaccompanied minors in 2011 and demonstrated insignificant positive differences with this group in the occurrence and level of depression and posttraumatic stress symptoms over time; however, there were some individual differences (Oppedal, Jensen, Brobakke Seglem, & Haukeland, 2011).

## 4 MATERNAL AND CHILD HEALTHCARE NEEDS IN NORWAY

### 4.1 Urban challenges

As in the United States (Woods, Hanson, Saxton, & Simms, 2016), immigrants tend to settle in specific areas of Norway. As an example, in the Stovner District of Oslo, the capital of Norway, 53% of the 32,000 inhabitants are immigrants or children of immigrant parents of mostly non‐Western background (95%) (Statistics Norway, 2016b). In fact, the residents represent over 142 countries. Since the 1970s, Stovner District has had alarming health and social statistics as compared to Norway in general. The district has the highest child poverty rate in Norway, as almost one in three children grow up in poor households (Municipality of Oslo, 2016) compared to every 10th child in 2000 (Nadim & Nielsen, 2009). Every third student drops out of high school, and 23% of the population over 20 years of age has no further education beyond primary school (Municipality of Oslo, 2016). Only 23% of the Stovner population has higher education, as compared to Oslo where 48% of the overall population has university or college education (Municipality of Oslo, 2016).

Over the last 10 years, Stovner District, and Norway in general, have experienced a substantial shift in allocation of funds due to increased use of Child Protective Services (CPS); the Stovner CPS now uses five times more resources as compared to the MCHS and the school health service combined (Stovner District, 2016). Statistics from CPS have shown, however, no overrepresentation of immigrant children or children of immigrant parent(s) (The Norwegian Board of Health Supervision, 2017). The increased cost within CPS is due to more complex cases and an increase in cases requiring follow‐up. Children served by (or under the care of) CPS have an increased risk for school dropout and later absence from work. For society, the long‐term consequences are costly (Vinnerljung & Sallnäs, 2008).

Recognizing the need to reverse and limit the human and financial costs associated with child protection issues and a desire to create a more equal society, several health policies were enacted to boost health prevention and promotion, including the Coordination Reform (2008–2009), the Public Health Act (2012), and the Act Relating to Municipal Health and Care (2012). Numerous international studies have demonstrated how interventions supporting the parent–child relationship, child development, and child social adaption lead to improved school readiness and may reduce costly secondary and tertiary interventions (e.g., Eckenrode et al., 2010; Owen‐Jones et al., 2013). Thus, municipalities responsible for managing the services within the PHCS have placed an emphasis on early health promotion and prevention, specifically focusing on supporting healthy socioemotional development and strengthening positive parent–child relationships (Norwegian Directorate of Health, 2017). In addition, there was a desire to stem the increasing medicalization of everyday problems and to adjust services to the individual child's health and development, consistent with the PHN value of promoting equality through universal services. As Andrews (2006) stated,

Instead of attempting to thrust standardized advice upon parents, professionals were recommended to take each family's different circumstances into account, to focus on problems as experienced by the parents themselves and to serve parents according to parents’ own definitions of their needs. (p. 192)

PHNs are educated to respect each family's boundaries and to respect their culture and method of rearing practices, but within the Norwegian laws. For instance, it is illegal to use physical/corporal punishment when disciplining children in Norway. Studies have indicated that parents in Norway, to a large extent, trust the MCHS as a source of knowledge on infant and child care because of the care provided by the PHNs (Andrew, 2006).

## 5 CREATING A NEW PROGRAM WITHIN THE EXISTING MCHS

In 2013 at Stovner District, the district administration invited leaders in health‐related positions and researchers from the University of Oslo to a series of think tank meetings (later, established as the steering committee for the pilot). The agenda was to discuss possible approaches on how to accommodate incremental service demands and the aforementioned new political guidelines. Leaders of the district wanted a new program to be integrated within the existing MCHS services.

### 5.1 Program philosophy: Equality and equity

Rapid changes in the population composition are often accompanied by an upswing in xenophobia, defined as attitudinal, affective, and behavioral prejudice toward immigrants and those perceived as foreign (Yakushko, 2009). Xenophobia leads to discrimination, differential treatment that can be conscious and subconscious interpersonal interactions between individuals as well as institutionally or structurally engrained, systemic practices. In the United States, recent studies have linked the impact of exposure to racism and discrimination to health outcomes such as cardiovascular disease, mental health problems, and poor self‐perceived health (Paradies et al., 2015). Furthermore, structural racism interacting with social determinants for health increases risk for myocardial infarction and adverse birth outcomes in minorities in the United States (Bailey et al., 2017; Lukachko, Hatzenbeuhler, & Keyes, 2014).

A recent systematic review has addressed how vicarious racism (secondhand exposure or observing racism) transmits trauma in children (Heard‐Garris, Cale, Camaj, Hamati, & Dominguez, 2018), and may be particularly relevant when caring for immigrant children and unaccompanied minors. In addition, implicit biases (attitudes or preferences that are automatic and occur without conscious control) often are exhibited in stressful situations. Implicit biases about certain groups exist independently of explicit beliefs and can influence not only social behavior but also medical and legal practices (Greenwald, McGhee, & Schwartz, 1998). Implicit bias, discrimination, and xenophobia may be a special challenge when caring for patients of different cultural backgrounds, language capabilities, and health literacy, and may be especially relevant when providing reproductive health services for immigrant populations (Hall et al., 2015). Balaam et al. (2013) found that migrant women in maternity care in Europe were concerned with preserving their integrity in the new country. Many struggled to find meaning, which was related to inadequate communication, lack of connection, striving to cope, struggling to ensure a safe pregnancy and childbirth, and maintaining bodily integrity. In a study of immigrant parents’ experiences with the Norwegian MCHS, some parents complained about being left without advice at the MCHS (Viken, Lyberg, & Severinsson, 2015).

Thus, in keeping with Norwegian healthcare service structure and the value of providing free healthcare for all in the MCHS system, and the consideration of the impact of racism and implicit bias on health and healthcare provision, the steering committee believed it was imperative that the program be available for *all* new families attending the MCHS in Stovner District, irrespective of background, immigrant status, nationality, or socioeconomic status.

### 5.2 Importance of the early years

The committee recognized that the first years of life are a critical time in a child's development (McCain & Mustard, 1999. Early experiences lay the foundation of physical, cognitive, language, social, and emotional development for future health and developmental progress (Wynder, 1998). By investing in interventions that support new families, it may be possible to prevent negative outcomes and promote positive and sustainable child development for the short‐ and long‐term (Armstrong, Fraser, Dadds, & Morris, 2000; Enoch et al., 2016; Olds et al., 1998). Furthermore, by attending to the child's and the family's needs and strengths, appropriate and culturally relevant support and service can improve the child's and family's subsequent outcomes (Coles, Cheyne, & Daniel, 2015).

### 5.3 A salutogenic approach

With close to 80% of the newborn children in Stovner District being born to non‐Western immigrant parents, it was important that the approach supported the immigrant's inclusion and integration into the Norwegian MCHS and that the service was tailored to the specific needs of the families served. For many of the families, prior adverse experiences were prevalent. To reach them without overly focusing on deficits and risks, the salutogenic theory was embedded within the program. The salutogenic perspective to healthcare provision offers an alternative philosophy to the traditional pathology and risk perspectives (Antonovsky, 1996). Salutogenesis interprets the state of health as a continuum, with complete (positive) health at one extreme and total absence of health at the other. Under this theory, the health state oscillates along this continuum throughout our lives. By avoiding the all‐consuming concern with risk factors or pathogens, the salutogenic orientation operates with a “healthy/dis‐ease continuum” (Antonovsky, 1996). The theory emphasizes that people need to understand their own lives and to be understood by others, that individuals are capable of managing situations themselves, and most important, that what they perceive as meaningful provides motivation to work toward their goals (Mittelmark & Bull, 2013). The focus is on resilience, strengths, and sense of coherence rather than deficits and risk profile. While many early intervention programs in the United States also incorporate a strengths‐based approach, programs also emphasize identification of risk both for identifying who is eligible for services and for identifying activities of the home visitor (Armstrong et al., 2000; Enoch et al., 2016). Furthermore, implementing a salutogenic model could ensure the intention and tradition of the Norwegian universal approach at the MCHS as being an equal and equitable service for all (Lindström & Eriksson 2006). Staff at the MCHS had yearlong experiences working with immigrant families, and were informed about implicit bias and given the opportunity to take an implicit bias test, prior to starting the program. The test was to better understand that our attitudes or preferences are automatic and occur without conscious control. No other measures were obtained. Future research is needed to examine how experiences and observation of xenophobia, discrimination, and racial bias impact health over the life course, particularly preconception, during pregnancy, and in childhood in immigrant populations.

### 5.4 Community participation in program development

Involvement of the providers and recipients of services was deemed essential to developing a program that would be salient to their needs and concerns. A qualitative pilot study, approved by the Regional Ethics Committee, was conducted to determine the perspectives of the professionals and participants to evaluate home visiting as a method for intervening. Focus groups and key informant interviews with users of the MCHS, the PHNs, administrators of the MCHS, and administrators of Stovner District were undertaken. The results from these focus groups and key informant interviews were analyzed along with literature reviews, Norwegian policy documents, and standards for the MCHS practice. Using an iterative formative research process, community participation, professional and community education, and critical reflection contributed to the development, implementation, and expansion of the existing MCHS program. The focus groups and the key informant interviews provided insight into how home visits compared to visits at the MCHS. Mothers who were users of the MCHS in Stovner described how the home visits, with the PHN as a guest, created a more equal power balance between the mothers and their PHNs, as compared to office visits. In addition, more time on home visits was viewed as an essential component. Knowing that they did not have to end the visit after the typical 20‐min office visit opened opportunities for these mothers to talk about topics that were difficult. As one mother described: “I don't wanna talk about things that perhaps make me cry, and then have to leave her office crying (the PHNs) and walk past all the mothers sitting outside in the hallway.” Similarly, the PHNs felt that the home visits offered a wider picture of the family; they could use “all their senses.” At the office, they only saw the parent and the child in an unfamiliar setting. At home, both the child and the parent(s) appeared more relaxed, which had a positive effect on the home visits. In addition, the PHNs could give more individualized guidance and advice when they knew and could see the home setting.

## 6 THE NEW FAMILIES PROGRAM

As a result of the processes described earlier, the New Families Program (The pilot was called *New Mothers project*.) was created as an early intervention initiative serving all first‐time families and their infants in Stovner District (Leirbakk et al., 2018). The aims of the program were to improve the parent–child relationship, child health and development, enhance parental self‐efficacy, children's social adaptation, and school readiness, with an overarching, long‐term health systems goal to reduce costly secondary and tertiary preventive measures. Specifically, the project was to provide increased home visits by PHNs during pregnancy and up until the child was 2 years of age, which would supplement the current services. PHNs would act as traditional “experts” as well as “enablers” to encourage parental independence and autonomy.

### 6.1 Home visits

As mentioned, Norwegian guidelines require the PHN to conduct one home visit to every woman within 2 weeks after delivery (Misvær & Lagerløv, 2013). International literature has demonstrated that more intensive home‐based interventions provided by nurses, aimed at high‐risk families during the first years of an infant's life, show promise in promoting child health, family functioning, and the subsequent use of welfare (Kendrick et al., 2000; Kitzman et al., 1997). Focus groups with MCHS users, both immigrants and Norwegian‐born in the New Families Program, were favorable to having more home visits.

In the New Families Program, PHNs from the MCHS schedule the first home visit after 28 weeks of pregnancy; the same PHN follows the infant and mother at the MCHS clinic visits. Often there are several home visits prior to birth, although the number of visits depend on the mother's needs and wishes, and at least one home visit within the first 2 weeks after delivery. Postnatally, PHNs offer home visits to provide education and health supervision on a needs basis until the child is 2 years old. During the MCHS visits, PHNs follow the Norwegian standardized, age‐anchored guidelines when providing physical assessment, immunizations, guidance about infant health and developmental needs and parenting practices, and addressing questions related to domestic violence and depression. If the PHN suspects any form of child maltreatment, she or he is required by law to report this to the CPS. PHNs are trained to recognize symptoms of maternal depression, especially postnatal depression, and may use the Edinburgh Postnatal Depression Scale (Cox et al., 1987) if depression is suspected. When depression symptoms are identified, the PHN can refer the parent to the GP or a psychologist. At MCHS visits, all information obtained is documented in the health record. In contrast, the PHN does not take notes or do systematic assessments during the home visits; however, comments related to the home visit are entered into the health journal when the PHN gets back to the office. Topics addressed during the home visits can include anticipations and dreams about the baby, the parent's experiences as a child, reflections about the birth, the experience of the birth, aspects related to parenting, parent–child communication, the child's way of communicating, collaboration with partner or other adults, availability of a social network, and aspects related to work life or returning to work, and so on. Any triggers or high‐risk factors within the family are explored further at a later home or MCHS clinic visit. Mental health services and psychology referrals are available at each MCHS, including infant mental health services. In addition, PHNs can discuss their cases with the MCHS mental health experts at regular mental health team meetings. Cases which warrant referrals typically include maternal sleep deprivation, maternal depression, behavior problems with the child/baby, or any kind of problem that the parents or the PHN believe require mental health expertise.

From March 2014 to March 2016, 84% of all first‐time families in Stovner District agreed to participate in the New Families Program. The PHNs expressed that the opportunity to visit at home creates a stronger relationship to the family and a greater understanding of the family's situation, which enables the PHN to target her support and services. Similarly, the PHN's presence in the home was well‐received and experienced as beneficial by the mother, the family, and the PHNs. The mothers disclose great confidence with the MCHS and a sense of increased inclusion (Leirbakk, Dolvik, & Magnus, 2017).

## 7 CHALLENGES AND LESSONS LEARNED

Limited knowledge of the Norwegian language is a barrier for the immigrants the first years after arrival. Those who do not speak Norwegian are entitled to an interpreter at all health encounters and also at the MCHS visits. However, this is often not preferred by the client, although this might cause a gap in the service delivered. In Norway, all MCHS visits are allocated a certain amount of time; for instance, 30 min for an 8th month healthy child consultation. Within these 30 min, PHNs are expected to give the same information and ask the same questions to a Norwegian‐speaking family as to a family where there is no common language, and this is challenging. In the New Families Program, time duration for a home visit is not limited, which enables the family and the nurses to find ways to communicate. This can be through using videos and/or pictures, and the time to get to understand each other is of great importance. Notably, many of the families want to exercise their limited Norwegian; thus, they often do not want an interpreter.

An additional challenge is low general literacy and limited education from their home country. In the New Families Program, PHNs generally do not include an interpreter at the first home visit. The following quote from one of the PHNs illustrates their sentiments:

Yes, you feel you are treading on them a little, when you ask for the presence of an interpreter. I have to say, ‘It is I who need an interpreter to talk with you. It is my need, not yours, it is not you who need it, it's I, so that I can talk to you.’ It is always a balance. They want to manage, they want to speak Norwegian. They want to succeed; they want to use their Norwegian.

Thus, the lack of perceived need for an interpreter reflects both the desire/motivation of new immigrants to learn the language and culture of Norway, and underscores a salutogenic philosophy approach that encourages autonomy and independence. The PHN conducts some visits at home without an interpreter, but might say that she or he wants an interpreter at the MCHS visits as they then will address all the issues, the “list” she or he has to complete at the clinic.

During the focus groups conducted in the initial phase of the development of the New Families Program to assess the feasibility of an increase in home visits, the global and most critical concern for all these mothers was related to not being a “good mother.” Recognizing this, the PHNs often include in their first visit questions about the mother's ideas of becoming a mother, such as “How do you feel about becoming a mother?” “What were your experiences as a child with women that were mothers?” During home visits, the PHN's traditional role as health expert becomes modified as she or he also becomes a visitor and mentor to the parent, establishing a different climate for the PHN–parent relationship. As the PHN relinquishes some of the power and the role as an “expert,” approaches the mother and the family with attentive (active) listening (Zapart, Knight, & Kemp, 2015), and focuses on resilience and strengths rather than the risk factors that might be glaring, something special emerges. During the pilot of the New Families Program, PHNs stated that conducting the home visits and participating in the development of the New Families Program changed their praxis and made their regular work at the MCHS better, changing the way of viewing their work. The strategies evolving during the New Families Program created a shift in PHNs’ mindset, from focusing on statutory responsibilities to recognizing the value of each mother's and family's knowledge and experience, underscoring the importance in creating trust to facilitate support of vulnerable families. PHNs also increased their awareness of individual family needs and their necessity to tailor services. By offering additional home visits to all first‐time families, PHNs recognized that low‐risk families also had needs; families who appeared to cope still needed and appreciated guidance on how to adapt to a new family situation. Some immigrants did not know what MCHS meant, and wondered if it was a free offer or a way for the government to monitor the family. Another important aspect was time to engage in the families’ needs. The additional home visits enabled the PHNs to ensure equal service to all families and allowed them to use the requested time to elaborate on the emotional and social needs unique to each family.

## 8 DISCUSSION AND IMPLICATIONS

The health and well‐being of immigrant mothers, infants, and families remain a challenge for countries with rapidly growing immigrant populations. While not all immigrants have poorer health, as compared to native populations, when they enter, at least in some countries health can deteriorate as immigrants assimilate into a new country (Diaz et al., 2017). On the other hand, many immigrants are at increased risk for health problems by virtue of poverty, stressors related to migration, language, literacy, and cultural differences, lack of access to health services, and discrimination. Across countries, immigrant women have reported system and attitudinal barriers that result in their reluctance, if not complete inability, to access preventive care or services, but the women also share similar desires in terms of the care that they receive (Small et al., 2014). These barriers are directly related to reproductive health and justice.

Several theoretical approaches have laid the foundation for the development of the New Families Program; these include “equity,” “salutogenic approach,” and “relationship building.” The Norwegian New Families Program shares many similar approaches to home‐visiting programs developed in the United States. It supports vulnerable new families by providing education, support, more intensive services characterized by home visiting and increased clinic visits, and continuity of care by a consistent, trustworthy nurse assigned to the family over a 2‐year plus period. It emphasizes supporting the parent–infant relationship by empowering the mother to better understand her own and her child's health, developmental, and social needs and to use services more effectively; it also provides parenting guidance and resources to minimize early emerging problems. The New Families Program is a promising approach for these vulnerable families and provides insight into how barriers can be addressed and support provided to increase access to reproductive and maternal healthcare options and facilitate empowered decision‐making for families.

Relevant to reproductive justice, a unique aspect of the New Families Program is the strong and intentional incorporation of principles of justice and equity into services. First and perhaps foremost, Norway's universal healthcare system and organization of healthcare is facilitating this. Second, the local health authority, the leadership at the MCHS, and PHNs in the New Families Program are able and willing to accommodate the needs of the individual MCHS client, including vulnerable populations, without stigmatizing. While the costs of healthcare for the country have increased greatly in the past few years (a similar concern for the United States) and the healthcare needs have changed at least in some communities, the overarching commitment to healthcare as a “national security responsibility” stands in contrast to the U.S. approach of optional, or for many immigrant women and children, prohibition of access to healthcare. Norwegian health priorities enable an open, accessible system that welcomes all citizens, including immigrants, and ensures the health of pregnant women, infants, and children regardless of immigrant status. Structurally, local control means that municipalities and districts can identify and address the community's healthcare needs in a more manageable, flexible, targeted, and perhaps more effective manner. Thus, the New Families Program can use the national and local values and priorities to execute a program that not only meets the broad health goals for all citizens but also is culturally salient to the new populations served.

Second, the salutogenic perspective, which considers the entire continuum of health, is embedded as the theoretical underpinning of the New Families Program. While risks and problems are not ignored, the emphasis on building on the strengths of the family and supporting its autonomy changes the relationship between the healthcare provider and the family, and acknowledges partnership and collaboration as a fundamental premise of healthcare. This theoretical underpinning is consistent with the current PHN education and training in Norway emphasizing health promotion and establishing positive partnerships with families, in a healthcare system that encourages citizen participation. This approach may help to decrease stigma about the health and social needs of immigrants and increase engagement in tailored services. Furthermore, by encouraging autonomy and effective use of the services available to them, an indirect effect may be that immigrants learn about the culture that they are joining, perhaps leading to a stronger sense of inclusiveness and acceptance into the larger society. Of note, while use of the salutogenic theoretical framework had not been applied in PCHS in maternal or child healthcare settings as was the MCHS when we initiated the development of the New Families Program, several recent studies have done so (Ferguson, Davis, Browne & Taylor, 2015a, 2015b; Ferguson, Browne, Taylor, & Davis, 2016; Perez‐Botella, Downe, Magistretti, Lindstrom, & Berg, 2015). Notably, some aspects of the salutogenic approach are indeed familiar to U.S. home‐visiting programs (e.g., emphasis on collaboration and positive partnerships between parent and professional, empowering autonomy in the parent, focus on health promotion). However, universal availability of healthcare for pregnant women, infants, and children regardless of immigrant legal status, the relative de‐emphasis of risk, and the explicit desire to facilitate immigrant families’ sense of inclusion and acceptance into the larger society provide a contrast to usual U.S. care.

Most salient to infant mental health is the emphasis of the New Families Program on relationship building. Preliminary qualitative data suggest that the PHN–mother relationship established in the New Families Program is transformative not only for the mother and the mother–infant relationship but also for the PHNs and the MCHS culture and work environment. There is better understanding of the needs of families and greater recognition of the capabilities and resources of the families, enhancing the professionals’ and the families’ abilities to address barriers that can interfere with healthcare and to take care of the infants in a safe, nurturing, and developmentally sound way. This is not new news for those who work with vulnerable families and their young infants, but it serves as an important reminder that “good care,” whether in a universal healthcare system or in a mixed private–public system such as in the United States, requires the development of trusting, dependable relationships with families as well as within systems and communities.

Finally, there is still much work to be done. While the New Families Program will be offered throughout all of the MCHSs in Oslo by the end of 2019, further research is needed to determine whether mothers, infants, and families who participate in the program indeed reach the maternal and child health and social outcomes set forth by the program, whether equitable access and care is achieved, and the cost–benefits of the program as compared to the usual care provided to the maternal–child population. Newly granted funds from the Norwegian Research Council will allow a case‐control study to follow up families over a period of time, and the study already has been initiated. Like Norway, healthcare in the United States is in a time of great transition, and health equity is one area of concern especially for migrant women, children, and families (AAP, 2005; ACOG, 2015; Lauen et al., 2017). Advocacy organizations such as the National Latina Institute for Reproductive Health, National Asian Pacific American Women's Forum, and Sistersong Women of Color Reproductive Justice Collective increase awareness of reproductive justice issues, identify systems and power structures that reinforce or challenge oppression of minority and migrant women, and advocate for healthcare policies that elevate and make visible the needs of marginalized women (and their children) and improve access to equitable healthcare in the United States. This knowledge is necessary to address needed change in the U.S. healthcare system, but we also can “look to Norway” to learn how healthcare systems can provide equitable, just, quality care to vulnerable young children and families.

## CONFLICT OF INTEREST

We have no conflict of interest to declare.
